# Enamel decussation pattern originates from directional sliding of ameloblasts

**DOI:** 10.1038/s41368-025-00412-5

**Published:** 2026-02-03

**Authors:** Vladislav Rakultsev, Josef Lavicky, Marcos Gonzalez Lopez, Klara Cigosova, Igor Adameyko, Jan Krivanek

**Affiliations:** 1 Department of Histology and Embryology, Faculty of Medicine, Masaryk University, Brno, Czech Republic; 2 Buchmann Institute for Molecular Life Sciences (BMLS), Institute of Cell Biology and Neuroscience, Goethe University Frankfurt, Frankfurt am Main, Germany; 3 Department of Neuroimmunology, Center for Brain Research, Medical University of Vienna, Vienna, Austria; 4 Department of Physiology and Pharmacology, Karolinska Institutet, Stockholm, Sweden

**Keywords:** Differentiation, Cell proliferation, Fluorescence imaging

## Abstract

Enamel, the inorganic tissue covering the crowns of teeth, is known for its remarkable resilience and hardness. These properties originate from its high proportion of mineralized matrix and complex internal microarchitecture. On an ultrastructural level, it consists of directionally arranged enamel prisms. Continuously growing rodent incisors are an exemplary case of this phenomenon. Their enamel has a consistent decussation pattern, providing teeth with extremely high resistance and ensuring they remain constantly sharp. While the decussation pattern has been described in detail, mechanisms behind its formation have not been experimentally proven. Here, we show that the highly organized enamel micropattern is generated by directional epithelial sliding of enamel-forming ameloblasts in vivo. Our results detail how enamel micropatterning stems from individual cell cluster segregation and subsequent reciprocal interweaving. Based on this determination, we introduce and experimentally demonstrate a new model of enamel decussation pattern formation.

## Introduction

Teeth are complex highly organized structures that serve multiple purposes, ranging from mastication to involvement in creation of certain sounds during vocalization. Teeth are comprised of hard tissues – enamel, dentin, and cementum – and contain a soft vascularized pulp on the inside, which is important for the maintenance, support and sensitivity of the tooth. The outermost part of the tooth, the crown (or crown analog), is usually covered by enamel. Enamel is the hardest mineralized tissue in the bodies of all vertebrates, containing more than 95% inorganic matter.^1^ Enamel serves to protect underlying dentin from external forces, pathogens, and chemicals, thus preventing infections and irritants from reaching the sensitive dentin-pulp complex.^2,3^ Although the hardness of enamel mainly derives from a high ratio of inorganic components, this is not the only contributing factor. Enamel’s high durability is also attained via an intricate inner enamel microarchitecture composed of an enamel prism system produced by ameloblasts—cells of an epithelial origin. During early odontogenesis, dental epithelium cells at the future positions of the teeth interact with the adjacent odontogenic ectomesenchyme and undergo orchestrated reciprocal signaling interactions. These interactions lead to the formation of preameloblasts, which further differentiate into enamel-producing ameloblasts.^4–8^ During this process, cells undergo several morphological and functional changes.^5^ The most drastic change happens during the transition to the secretory stage of ameloblasts, when preameloblasts acquire the ability to secrete enamel matrix proteins that later serve as a backbone for definitive enamel. During this stage, ameloblasts develop Tomes’ processes, through which they secrete matrix proteins, and change their apical-basal polarity.^9–11^ Significantly, every ameloblast, having only one such process, therefore builds a single enamel prism. As ameloblasts continue to secrete matrix proteins and initiate their mineralization, a range of complex enamel microstructure patterns is formed.

The enamel microstructure is species-dependent and has significantly changed throughout evolution. Amphibians and most reptiles have an enamel layer that contains no enamel prisms.^12^ This aprismatic enamel consists of specific enamel matrix proteins, yet has no higher organization. The more sophisticated prismatic enamel is believed to have first developed in mammalian species as a response to a more diverse diet and eating habits.^12,13^ In this type of enamel, the enamel prisms (often also referred to as enamel rods) are arranged in a precise, species-specific manner. In certain species, enamel prisms mutually interweave and create a highly organized enamel microstructure known as decussation patterns.^10,14–18^ An increase in the complexity of enamel prism organization can be observed throughout mammalian evolution, with decussation patterns present in additional parts of the enamel layer at increased prism tilt angles.^12,19^ This patterning is believed to be the basis behind enamel layer resilience, since it prevents cracks caused by mechanical forces from spreading deeper into the more delicate underlying dental tissues.^3,20–23^ This belief is further supported by the fact that in human teeth, areas with more pronounced enamel decussation are found on occlusal surfaces exposed to a higher mechanical load.^13,24^

The most striking case of intricate enamel microstructure can be found in rodents. These animals characteristically have continuously growing incisors (also other teeth in some species), providing them an evolutionary advantage in the processing of a highly abrasive diet.^25,26^ The thin layer of enamel, exclusively positioned on the labial aspect of the incisor, exhibits unique mechanical properties mainly stemming from the highly organized enamel prisms. Their enamel prisms are organized in sheets with alternating tilt angles, creating a remarkably regular decussation pattern specifically characteristic for the inner enamel layer.^14,18,23,27,28^ Interestingly, the formation of the decussation pattern has been shown to be influenced by tooth wear, which is related to the animal’s dietary habits and biting force.^29^ Despite the extensive research conducted to understand the origin of the enamel decussation pattern, precise mechanisms behind its formation remain poorly understood.

With this study, we took advantage of the decussation pattern model found in the continuously growing mouse incisor. Using single-cell RNA sequencing (scRNA-seq) approaches, several groups have recently determined that, in mouse incisors, the ameloblast-producing labial epithelial cervical loop (LaCL) is highly compartmentalized, and they suggested the existence of different mechanisms of epithelial turnover and maintenance.^30–32^ These large datasets, comprising different cell types and cell states, provide a unique opportunity to study the stem cell niche and mechanisms of turnover of dental epithelium on both cellular and transcriptional levels.

In this paper, we have identified and experimentally confirmed the existence of a new rare population of dental epithelial progenitor cells, characterized by the expression of *Sox10*, as previously suggested by scRNA-seq data.^30^ Using genetic lineage tracing techniques and advanced confocal and light sheet timelapse microscopy, we reveal that *Sox10*+ dental epithelial progenitor cells uniquely reside only in the LaCL and not the lingual cervical loop (LiCL) of mouse incisors, and subsequently give rise to ameloblasts. These rare cells form a stable cell population, which resides in this region in a long-term scale, and continuously gives rise not only to new generations of ameloblasts, but also to other cell types in order to replenish other parts of the LaCL. With our approach, we were able to trace the fate of individual cells, which eventually formed spatially distinct, color-coded clusters of ameloblasts during the formation of the enamel decussation pattern. Finally, we demonstrate that *Sox10*+ cell progeny is capable of migration between distinct layers of the incisor’s LaCL.

Conclusively, we allude to a new mechanism of enamel decussation pattern formation using the mouse incisor model. Our results indicate that controlled cell division and directed migration of dental epithelial layers of (pre)ameloblasts stand behind the formation of enamel decussation pattern. Our results also suggest that the plasticity of the cell niche residing in the epithelial loop of the tooth has a more complex hierarchy than previously anticipated.

## Results

### Identification of rare *Sox10*+ dental epithelial progenitor cells in mouse incisors

**Fig. 1:**
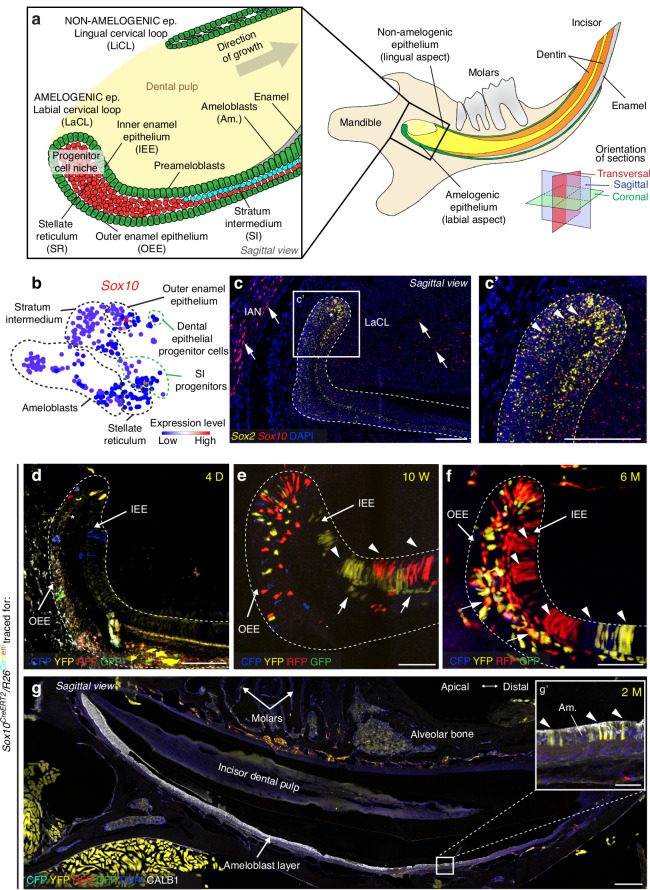
Identification of *Sox10*+ amelogenic dental epithelial progenitor cells in continuously growing teeth. **a** Schematic drawing showing a sagittal section of a mouse mandible which contains the continuously growing incisor, with an inset showing details of the stratification of dental epithelium in the apical portion of the incisor. Colored squares represent orientations of histological sections. **b** Mapping of cell populations identified by the expression of *Sox10* in datasets available from.^30^ Circles indicate populations of dental epithelial progenitor cells and stratum intermedium (SI) progenitors, with a limited number of cells in these populations expressing high levels of *Sox10*. **c,** **c’** In situ hybridization shows co-expression of *Sox10* and *Sox2* within the labial cervical loop (LaCL) region (asterisk), shown in detail in (**c’**). Arrows point towards *Sox10*+ glial cells in the inferior alveolar nerve (IAN) and pulp, arrowheads indicate *Sox10*+ cells inside the LaCL. **d**–**f** Lineage tracing of *Sox10*+ progenitor cells in incisors of the *Sox10*^*CreERT2*^*/R26*^*Confetti*^ mouse strain depicts lineage-traced cells in the stellate reticulum (asterisks) on sagittal tissue sections. When traced for 4 days (4D) (**d**), labeled cells are present exclusively in the most apical part of the LaCL. When tracing duration is extended to 10 weeks (10W) (**e**) and 6 months (6M) (**f**), traced cells are present in the entire LaCL, layers of preameloblasts and ameloblasts (arrowheads). Traced cells can also be observed in the SI (arrows). **g**, **g’** Co-staining of *Sox10*^*CreERT2*^*/R26*^*Confetti*^ mouse incisors with an anti-*CALB1* antibody shows lineage-traced mature ameloblasts. See also Fig. S1. Scale bars: 50 μm in (**c**–**f**), (**c’**) and (**g’**), 250 μm in (**g**). (Am. – ameloblasts, D – days of lineage tracing, Ep. – epithelium, IAN – inferior alveolar nerve, IEE – inner enamel epithelium, LaCL – labial cervical loop, LiCL – lingual cervical loop, M – months of lineage tracing, OEE – outer enamel epithelium, SI – stratum intermedium, SR – stellate reticulum, W – weeks of lineage tracing)

Our previous scRNA-seq data suggested the existence of an intricate hierarchy of the dental epithelial progenitor cell niche in continuously growing mouse incisors (Fig. 1a).^30^ To uncover the stemness potential of one of these very rare cell clusters, we selected *Sox10* as a marker gene, whose expression was specifically upregulated in a very limited number of cells clustered within the expected progenitor area (Fig. 1b). To validate the location of the *Sox10*+ region within the LaCL, we performed an in situ hybridization and co-detected *Sox10* with *Sox2*, a well-established marker of dental epithelial stem cells.^33–36^ *Sox10* exhibits scattered expression within the LaCL and is co-localized with *Sox2*, which suggests that *Sox10*+ cells could possess stem cell properties (Fig. 1c). To confirm the differentiation potential of *Sox10*+ epithelial cells, we performed lineage tracing using the *Sox10*^*CreERT2*^*/R26*^*Confetti*^ mouse strain (Fig. 1d–g). Short-term (4 days) lineage tracing uncovered a low number of lineage-traced cells located exclusively in the most apical part of the LaCL, including the early inner enamel epithelium (IEE) and outer enamel epithelium (OEE) and the stellate reticulum (SR) (Fig. 1d). Yet, no lineage-traced cells were observed among ameloblasts. Conversely, the long-term lineage tracing experiments (from 10 weeks to 6 months) show the presence of an expanding population of lineage-traced cells in the LaCL, including the late stages of OEE, remaining parts of SR, and, most importantly, preameloblasts and ameloblasts (Fig. 1e,f; Fig. S1). Completed differentiation of lineage-traced *Sox10+* cells into functional enamel matrix-producing *CALB1*+ ameloblasts is shown in Fig. 1g. These results showcased the nature of *Sox10+* dental epithelial population and its differentiation potential into functional enamel-producing ameloblasts. Notably, lineage tracing using the *Sox10*^*CreERT2*^*/R26*^*Confetti*^ construct is designed to label only a limited number of cells (Fig. 1d), providing the opportunity to follow the separate cell clusters originating from a single lineage-traced *Sox10+* progenitor cell.

**Fig. 2:**
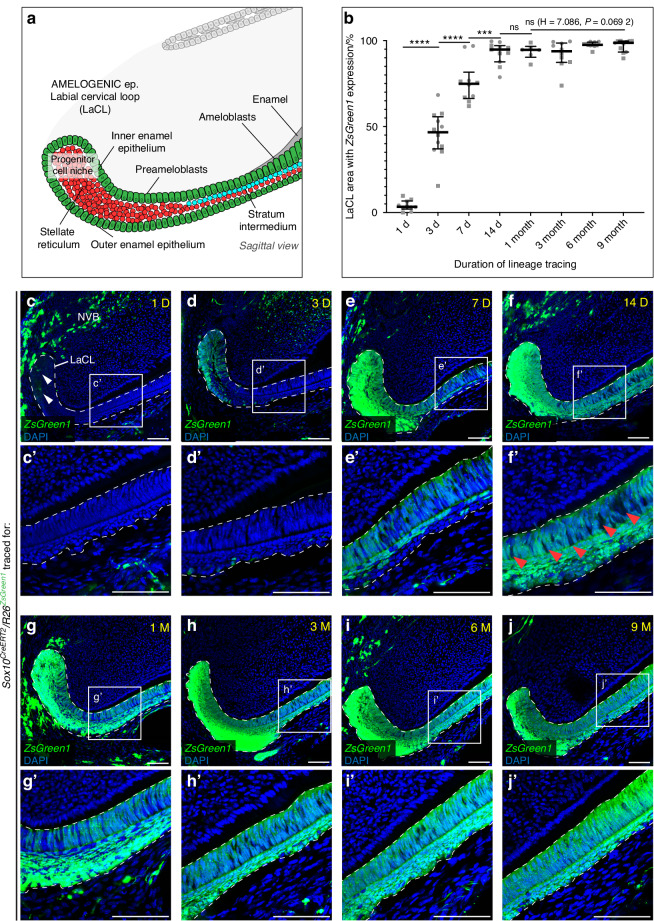
Expansion of *Sox10*+ cell progeny in the amelogenic epithelium. **a** Schematic drawing shows the cellular organization of dental epithelium in the labial cervical loop (LaCL) of mouse incisors in a sagittal view. **b**–**j** Graph (**b**) and confocal microscopy images of 14 μm sagittal sections of *Sox10*^*CreERT2*^*/R26*^*ZsGreen1*^ mouse incisors (**c**–**j**) show the expansion of lineage-traced cells (arrowheads) in the LaCL of incisors over time. Lineage-traced *ZsGreen1*+ cells rapidly expand within the LaCL (**c**–**f**), reaching maximum coverage ( > 90%) of the analyzed area after 14 days of tracing. Traced cells remain resident in the LaCL even after 9 months of lineage tracing (**g**–**j**). **c’**–**j’** Detailed sagittal views of traced cells in the dental epithelium reveal that traced cells are present in the ameloblast layer, the stratum intermedium, the stellate reticulum and the outer enamel epithelium. Arrowheads mark regions of the ameloblast layer where no *ZsGreen1*+ cells are found, showing that the recombination of the reporter construct is not absolute. Whiskers above and below the boxes in (**b**) indicate the maximum and minimum values, horizontal lines show the median value. N: 6-13 sections from two mice evaluated for each time point (values from each of the individual mice are shown as different symbols in all timepoints). See also Figs. S2, S3. Scale bars: 30 μm. (D – days of lineage tracing, Ep. – epithelium, H – Kruskal–Wallis test statistic, LaCL – labial cervical loop, M – months of lineage tracing, ns – non-significant, NVB – neurovascular bundle, ***P < 0.001, ****P < 0.000 1)

To enable the quantification of the *Sox10*+ progeny at different timepoints, we used the *Sox10*^*CreERT2*^*/R26*^*ZsGreen1*^ mouse strain, which has a higher reporter recombination rate (Fig. 2). Two biological replicates (two different mice) of identical genotypes were produced for each time point of the experiment. From each mouse, consecutive sections of one mandibular incisor were produced. In total for each biological replicate, at least five consecutive sections were analyzed for each time point. To increase the consistency of measurements, images of sections that were not produced in an optimal sagittal plane were excluded from further evaluation. The lineage-traced *ZsGreen1*+ cells rapidly expanded within the LaCL, reaching maximum coverage ( > 90%) of the analyzed area after 14 days (Fig. 2a–f; Fig. S2). Coverage remained at this level even after long-term (9 months) lineage tracing, which is the time period during which mouse mandibular incisor completely renews 5-6 times (Fig. 2b–j).^37^ Essentially, lineage-traced cells in both *Sox10*^*CreERT2*^*/R26*^*ZsGreen1*^ and *Sox10*^*CreERT2*^*/R26*^*Confetti*^ mouse lines remained present and active in the LaCL even after long-term (9; 12 months) of lineage tracing (Fig. 1f; 2b–j).

Concurrently, we confirm that during short-term lineage tracing, only few cells in the LaCL are traced, while later on a large proportion of cells in LaCL (or virtually the whole population in case of *Sox10*^*CreERT2*^*/R26*^*ZsGreen1*^) is lineage traced (Fig. 1d–f; Fig. 2). The increasing number of lineage-traced cells in LaCL after a long period of lineage tracing suggests a possible transition between rare *Sox10+* progenitor cells and other sources of more abundant *Sox10-* cell types, signifying a high cellular plasticity of this region.^38^ In contrast to the situation in the LaCL, the analysis of the non-amelogenic LiCL has not shown any lineage-traced cells in this region even after long-term (9 months) tracing, pointing to an exclusive presence of *Sox10*+ progenitor cells in the amelogenic LaCL (Fig. S3). Altogether, our results prove the differentiation capacity of *Sox10+* cells, which are specific for the amelogenic dental epithelium. Lineage tracing of this rare cell type using the *Sox10*^*CreERT2*^*/R26*^*Confetti*^ mouse line enabled the tracking of separate, color-coded clones of (pre)ameloblasts during the formation of the enamel decussation pattern.

### Lineage tracing of clonal ameloblast clusters uncovers the mechanism of enamel decussation pattern formation

**Fig. 3:**
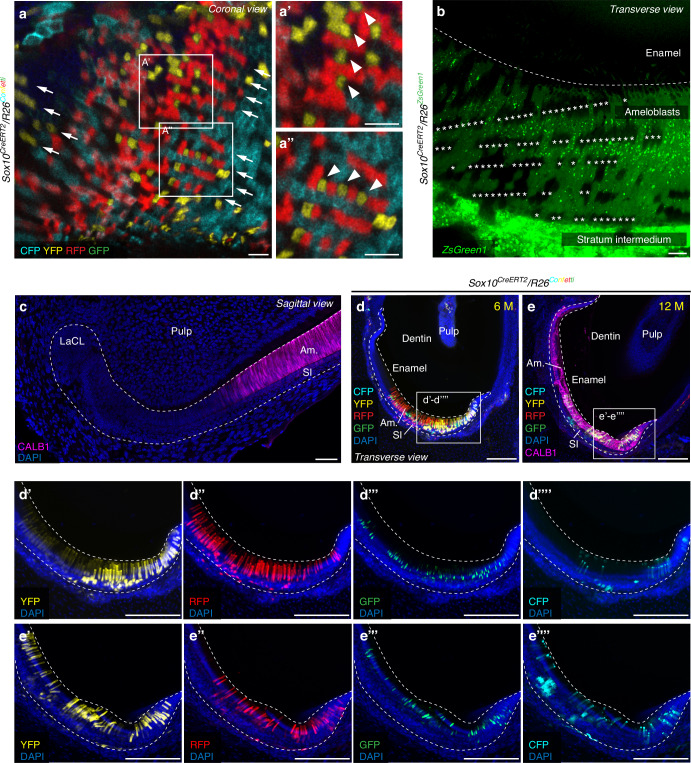
Clonal clusters of ameloblasts contribute to the formation of the enamel decussation pattern. **a** Lineage tracing of *Sox10*+ cells in the dental epithelium of incisors of the *Sox10*^*CreERT2*^*/R26*^*Confetti*^ mouse strain on 150 μm coronal sections. Single-layer stacks of ameloblasts (marked with arrows) regularly interweave with stacks of clones labeled in a different color, showing the periodicity during the initiation of enamel pattern formation. **a’**, **a”** Detailed view of lineage-traced ameloblasts shows the intricate alternating pattern (arrowheads). **b** Lineage tracing of *Sox10*+ cells in the dental epithelium of incisors of the *Sox10*^*CreERT2*^*/R26*^*ZsGreen1*^ mouse strain on a 50 μm transverse section reveals stacks of traced cells interweaving with stacks of non-traced cells (asterisks). **c** Immunostaining with an anti-*CALB1* antibody shows the beginning of a secretory-stage ameloblast cell layer, 14 μm section. **d**, **e** Long-term lineage tracing on 300 μm oblique transverse sections of *Sox10*^*CreERT2*^*/R26*^*Confetti*^ mouse incisors show non-clustered ameloblasts, where cells labeled with different fluorescent proteins are combined together in one region (shown in detail in (**d’**)–(**d””**) and (**e’**)–(**e””**)), suggesting the blending of the clonal progenies. Section (**e**) was additionally immunostained with an anti-*CALB1* antibody to label the ameloblast cell layer. See also Fig. S4. Scale bars: 10 μm in (**a**), (**a’**) and (**b**), 50 μm in (**c**), 150 μm in (**d**), (**d’**)–(**d””**), (**e**) and (**e’**)–(**e””**). (Am. – ameloblasts, LaCL – labial cervical loop, M – months of lineage tracing, SI – stratum intermedium)

**Fig. 4:**
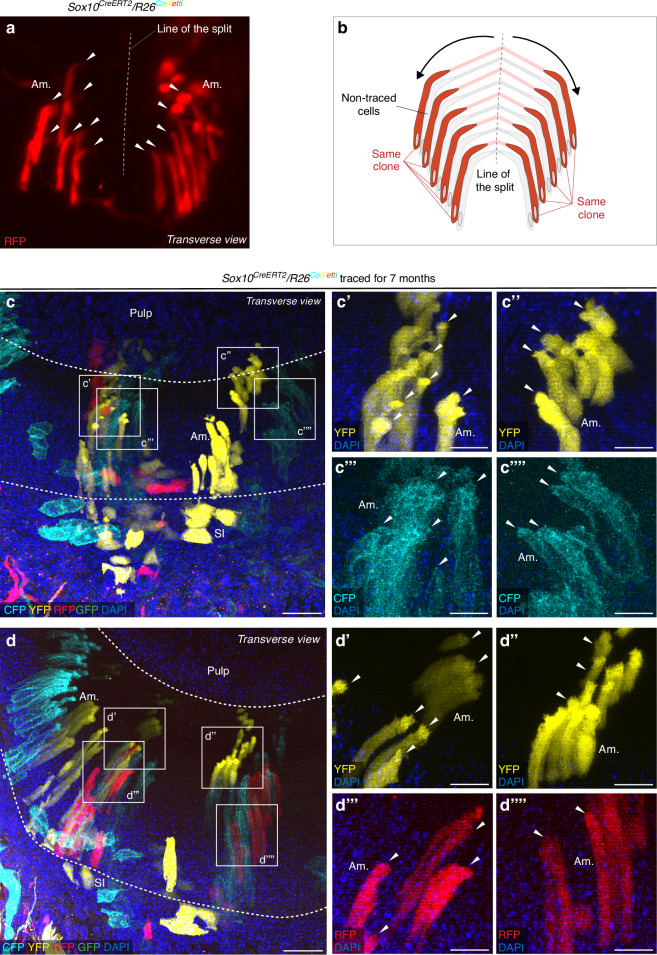
Spatial distribution of clonal clusters of ameloblasts defines the origin of the enamel decussation pattern. **a** Whole-mount light sheet image of a single cluster of ameloblasts on the surface of *Sox10*^*CreERT2*^*/R26*^*Confetti*^ mouse incisors shows ameloblasts originating from a single progenitor cell (based on the expression of RFP) migrating away from the line of the split. Note how Tomes’ processes (arrowheads) of ameloblasts are oriented towards the starting point of directed cell migration – the line of the split. **b** Schematic drawing of cells seen in (**a**) depicts the proposed explanation of the origin of the decussation pattern: ameloblasts residing along the line of the split migrate further away from the line in opposite directions. **c**–**d** Confocal microscopy images of 50 μm sections of lineage-traced *Sox10*^*CreERT2*^*/R26*^*Confetti*^ mouse incisors in a transverse plane show ameloblasts labeled with fluorescent proteins split into similarly sized groups. **c’**–**c””** and **d’**–**d””** Detailed view of grouped ameloblasts shown in (**c**) and (**d**), expressing a single shared fluorescent protein per group. Note how Tomes’ processes (arrowheads) of ameloblasts are oriented towards the point of origin of respective cells. See also Fig. S5. Scale bars: 10 μm. (Am. – ameloblasts, SI – stratum intermedium)

Using the *Sox10*^*CreERT2*^*/R26*^*Confetti*^ mouse strain, we characterized the clonal contribution of *Sox10*+ epithelial progenitor cells to ameloblasts. We took advantage of the *Confetti* reporter to label single progenitor cells and analyzed their clonal contribution during enamel decussation pattern formation (Figs. 3a,d,e, 4, S4). Sparse recombination of the *Confetti* reporter enabled tracing of the progeny originating from just several individual *Sox10+* cells, and thus allowed to follow their fate among other non-traced or differently color-coded traced cell progenies. On coronal sections, such lineage-traced ameloblasts show an alternating pattern, where ameloblasts of a different origin (RFP- and YFP-labeled) are systematically interchanged within a single layer of ameloblasts (Fig. 3a). This arrangement of fluorescently labeled ameloblasts mirrors the arrangement of enamel prisms seen in decussation patterns. Moreover, characteristic single-layer stacks of ameloblasts can be identified (Fig. 3a). These stacks originate from the same cell clone and are regularly interchanged with stacks of clones labeled by a different fluorescent reporter, displaying periodicity during enamel pattern formation initiation (Fig. 3a). A similar pattern was also observed when the single-color *ZsGreen1* reporter was used. Here, a different parameter (arrangement of *ZsGreen1*- cells) was followed, since a high, but not absolute, recombination of the reporter construct was observed. This enabled identifying the presence of regular non-traced stacks of cells alternating with lineage-traced cells (Fig. 3b). Furthermore, long-term *Confetti* lineage tracing on the sagittal sections indicated that ameloblasts labeled with different fluorescent proteins were combined together, suggesting a complex shuffling of different color-coded clones (Figs. 3c–e, S4).

Additionally, to follow the early process of enamel decussation patterning, high-resolution confocal images of thick transverse sections obtained from apical parts of *Sox10*^*CreERT2*^*/R26*^*Confetti*^ mouse incisors were analyzed (Fig. 4). These results imply that an early mechanism of decussation pattern formation lies in an equal split of ameloblast clusters, which originate from the same progenitor cell clone (and thus possess identical rare color codes) (Figs. 4; S5). Observed groups of neighboring cells that express the same fluorescent protein originated from a common progenitor cell. These almost equally split groups of same-colored cells divided into two parts, with both parts having Tomes’ processes pointing to the direction they originated from. Taken together, our results show the dynamics of the clonal origin of the early ameloblast arrangement. Furthermore, we show that the decussation pattern is formed by a complex interweaving of these cell clones as a result of a specific coordinated movement of individual cells within the amelogenic dental epithelium.

### Enamel decussation pattern originates from the directed migration of clonal clusters of ameloblasts

**Fig. 5:**
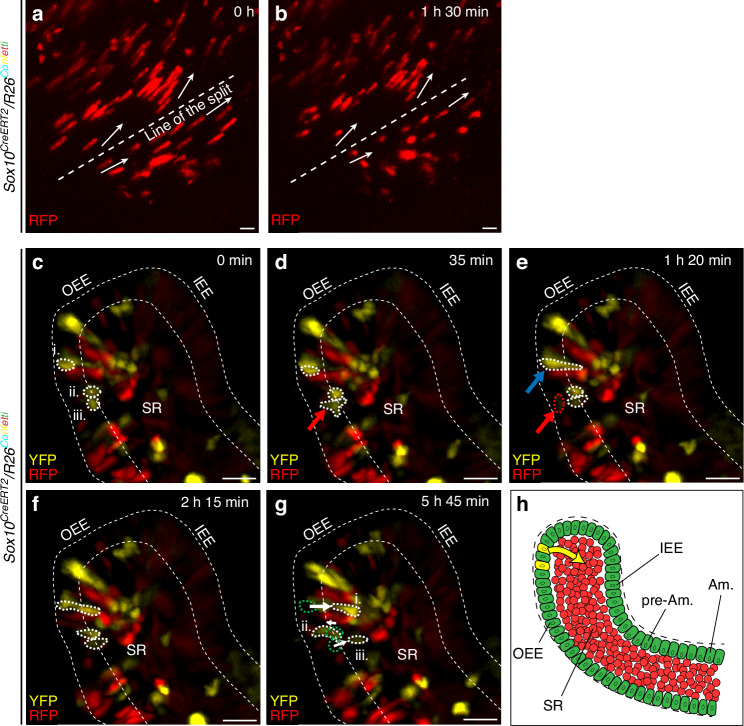
Live imaging of ameloblast movements and cellular plasticity within the labial cervical loop. **a**–**b** Live imaging of explants of *Sox10*^*CreERT2*^*/R26*^*Confetti*^ mouse incisors details ameloblasts migrating in clusters, with some groups of cells migrating in opposite directions (arrows) away from the line of the split (dashed line). **c**–**g** Live imaging of the labial cervical loop of *Sox10*^*CreERT2*^*/R26*^*Confetti*^ mouse incisors shows cells migrating from the outer enamel epithelium (OEE) layer into the stellate reticulum (SR). Initial positions of migrating cells (i., ii., iii.) are illustrated using dashed lines in (**c**). Arrow in (**d**) indicates a cell undergoing cell division, which is completed in (**e**). Blue arrow in (**e**) marks an elongated cell, which starts to migrate into the SR (**e**–**g**), while the red arrow and red dashed line mark the original cell that was seen dividing in (**d**), which created a migrating daughter cell. Arrows in (**g**) denote the direction of cellular migration, green dashed lines indicate the initial position of cells (i., ii., iii.) at the start of live imaging, white dashed lines determine the final position at the end of the experiment. **h** Schematic representation of migrating cells (arrow) within the labial cervical loop. See also Supplementary Movies S1 and S2. Scale bars: 20 μm. (Am. – ameloblasts, IEE – inner enamel epithelium, OEE – outer enamel epithelium, pre-Am. – preameloblasts, SR – stellate reticulum)

**Fig. 6:**
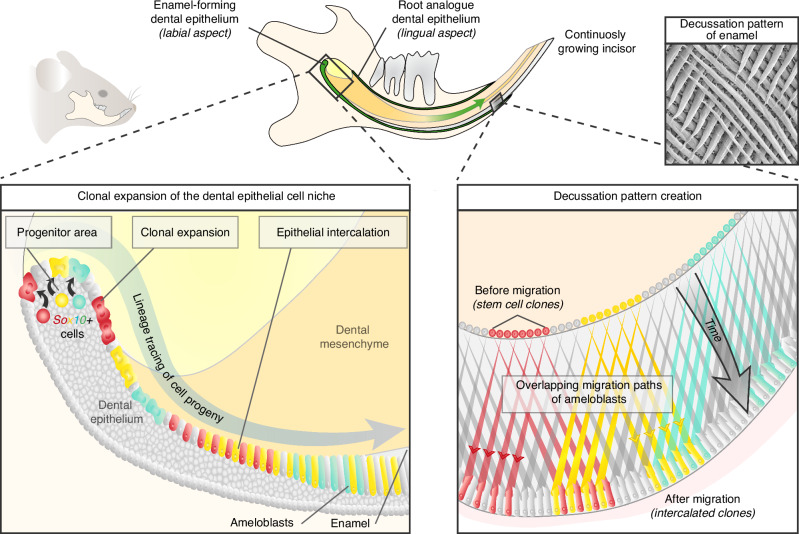
Summary of the proposed new model of enamel pattern decussation formation. Schematic drawing summarizing the suggested decussation pattern formation model. Upper drawing illustrates a sagittal section through the mouse mandibular incisor. Lower left panel shows how *Sox10*+ progenitor cells of the dental epithelium contribute to the ameloblast lineage, and how their progeny intercalates with *Sox10*- cells. Lower right panel schematically depicts the organized ameloblast migration along overlapping paths, which results in the formation of enamel decussation patterns (shown in the panel above)

Eventually, to show the dynamics of ameloblast movement during enamel patterning, we performed a 3D light sheet live imaging of *Sox10*^*CreERT2*^*/R26*^*Confetti*^ lineage-traced adult mouse incisor explants (Fig. 5a,b; Supplementary Movie S1). The results show that ameloblasts migrate as oriented cell clusters, moving in opposite directions away from the center axis. These findings further support our single-timepoint results illustrated in Fig. 4. Therefore, we hypothesize that populations of preameloblasts originating from the same clone are initially clustered together along the future *“line of the split”* – a supposed line, running parallel to the longitudinal axis of the incisor. Afterward, in a synchronized manner, they migrate away from this line in opposite directions, with each ameloblast leaving behind a single enamel prism. While migrating, ameloblasts of different clonal origins likely interweave, producing a complex arrangement of reshuffled ameloblast clones. Together, these events result in the formation of the enamel decussation pattern (summarized in Fig. 6).

Finally, to address the complexity of dynamics of the *Sox10+* cells and their early progeny, live imaging focused on the LaCL was performed (Fig. 5c–h, Supplementary Movie S2). The results revealed that some of the lineage-traced cells were capable of leaving the OEE layer and invading the SR of the incisor LaCL. Moreover, the lineage tracing also shows non-migrating cells, as well as cells that migrate in the opposite direction, from the SR toward the enamel epithelium. Together, these findings showcase another level of unanticipated cellular plasticity of the enamel-forming dental epithelium (Fig. 5c–h).

## Discussion

Although initial studies introducing the enamel decussation pattern appeared around half a century ago, the developmental processes leading to the acquisition of this remarkable ultrastructural feature, which provides teeth their characteristic resilience, remains unresolved.^14,15^ In this study, we suggest a new mechanism of the enamel decussation pattern formation via orchestrated directional migration of ameloblasts within the continuous epithelial layer. We took advantage of a multi-color genetic lineage tracing approach, targeting newly discovered rare dental epithelial progenitor cells. Using 3D confocal and light sheet timelapse imaging, we show how the splitting of individual neighboring ameloblast clones, followed by their orchestrated movement, results in their mutual interweaving. This work provides the first experimental proof that the enamel decussation pattern arises from continuous epithelial cell rearrangements. Moreover, it is likely that a similar mechanism as described in this study may also be responsible for the formation of Hunter-Schreger bands observed in other species (e.g., human teeth). These bands are regarded as an optical effect arising from differences in light reflection caused by distinct orientation of grouped enamel prisms.^13,39^

Many molecular markers of dental epithelial stem cells have been described in the past decades (reviewed in refs. ^26,38^). Challenging methods used to uncover stem cell characteristics have fundamentally advanced in recent years, owing to the emergence of scRNA-seq approaches.^30–32,40^ By reanalyzing a previously obtained scRNA-seq dataset of mouse incisors,^30^ we hypothesized and experimentally validated transcription factor *Sox10* as a new marker of a very rare progenitor cell type with potential to renew the whole LaCL of the incisors. The observed expression of *Sox10* in LaCL was co-localized with *Sox2*, a well-established marker of dental epithelial stem cells,^34^ indicating possible stemness of *Sox10*+ cells. Consistently, a co-expression of *Sox10* and *Sox2* has also been previously reported in other epithelial progenitors in exocrine glands.^41^ Stemness properties of *Sox10+* cells are recognized from many other tissues. In addition to *Sox10* being a well-known marker for Schwann cell precursors, *Sox10* is also involved in various epithelial tissue patterning.^42–49^ Interestingly, *Sox10* was recently indicated to be necessary for the transition of neural crest cells into their migratory state. Furthermore, *Sox10* has been implicated in promoting the migration of neural crest cells of the enteric nervous system via direct interaction with the *Cdh19* promoter, which affects the actin cytoskeleton and, in turn, influences cell migration.^50,51^ However, while our results suggest a stem cell potential of *Sox10*+ dental epithelial cells, we do not claim definitive evidence of stemness. As the main focus of this article was to describe the mechanisms of enamel micropatterning, we did not perform functional analyses to confirm their stemness. Additional studies are necessary to clarify the prospective stem cell properties of this cell population.

Although never previously proven experimentally, all earlier enamel micropatterning studies share a common idea: the coordinated migration of ameloblasts is crucially involved in the formation of the intricate enamel microstructure.^15,16,23,52,53^ While ameloblasts, as epithelial cells, are generally considered to be non-migratory, several recent publications have highlighted the importance of epithelial movements in various tissue patterning processes. The striking example of *Drosophila* hindgut formation, during which the epithelial structures rotate 90° counterclockwise, showcases a very important cell migration mechanism—cell sliding.^54^ In cell sliding, groups of cells move together in one direction without rearranging their junctions. The driving force behind such migration is cell asymmetry. At the start of migration, the cells exhibit left-right asymmetry, and later start migrating to finally become symmetric. A brief introduction of cell asymmetry is sufficient to induce cell sliding. Another mechanism important for epithelial morphogenesis is cell intercalation. Cell intercalation is an autonomous mechanism which requires Myosin II contractions and, similarly to cell sliding, can be induced by left-right cell asymmetry, leading to unidirectional cell movement, as seen in *Drosophila* genitalia rotation.^55^ In contrast to cell sliding, intercellular junctions are remodeled during cell intercalation.^55,56^ Numerical simulations suggest that the lengths of cell junctions can fluctuate over time, which triggers cell rearrangements. These fluctuations are caused by contractions of cell boundaries and, combined with the effects of planar cell polarity proteins, can bring about a unidirectional movement of cells while maintaining the integrity of the epithelial cell sheet.^57^ Rearrangements of epithelial cells are also common in vertebrates during epithelial morphogenesis and tissue regeneration, which are achieved by collective cell migration. This migration mechanism is characterized by the maintenance of junctional cell contacts, occurs actively in response to molecular cues, and is enabled via the force generated by the actin cytoskeleton.^58^ A well-described example of this mechanism is observed in mammary gland postnatal development, where cells of several types migrate in groups to shape the branched tissue.^59,60^

In the context of epithelial rearrangements during amelogenesis, there are several proposed mechanisms explaining ameloblast movements where: (1) the migration of rows of ameloblasts is an active process of cells themselves, based on cues not yet completely understood,^15,16,52^ (2) ameloblasts move passively owing to limited space in the extracellular matrix, where they deposit the enamel matrix,^15,16^ (3) the positions and trajectories of migrating ameloblasts are related to their spatial arrangement along the dentin-enamel junction at the early secretory stage of amelogenesis; subsequently, the ameloblasts migrate along divergent linear paths away from the starting point, which ultimately forms the decussation pattern with final ameloblast neighbor relationships differing from their initial arrangement,^23^ or (4) migration trajectories of ameloblasts depend on the balance between the intercellular mechanical strain and the initial intercellular distances in a row of migrating cells.^53^

Our results imply that the mechanism standing behind the enamel decussation pattern formation resembles cell intercalation, followed by cell sliding. Cell bodies of ameloblasts seem to first intercalate to initiate the decussation pattern formation, and then continue migrating by sliding as rows or sheets of cells while the enamel layer is being formed. Ameloblast migration is likely conditioned by certain factors needed for cell sliding, cell intercalation, or collective cell migration. The importance of cell junctions and actin cytoskeleton as the driving force of cell migration has already been indicated in the context of ameloblasts.^61^ Interestingly, significant differences in the decussation (or similar) patterns of different mammalian species have been described.^62,63^ Moreover, in a recent review, authors identified forty-one different genes as being responsible for enamel micropatterning.^64^ Altogether, this signifies that enamel prism patterning is a highly orchestrated and evolutionarily important event. Nevertheless, the molecular signaling driving the observed cell sliding (or other similar) ameloblast intraepithelial movement has not been elucidated yet.

Finally, our live imaging experiments establish that OEE cells can migrate into the SR layer and possibly contribute to its maintenance. This expands the current model of stem and progenitor cell homeostasis within the LaCL, which proposes that only IEE cells can contribute to SR maintenance.^31,38,65^ We therefore update this model by showing that the relationships of cells of different layers within this system are more plastic than previously believed.

In conclusion, our results explain and, for the first time, experimentally substantiate a mechanism for the origin of the enamel decussation pattern, driven by organized epithelial cell intercalation followed by epithelial cell sliding. More specifically, we propose that populations of ameloblasts reside along a line, which likely runs parallel to the longitudinal axis of the incisor enamel layer, and later undergo cell division, resulting in a cell lineage split and cell progeny migration away from this line in opposite directions. Such migration trajectories would permit the overlapping of migrating ameloblasts, which would then allow them to secrete enamel matrix in a manner that results in decussation pattern formation. Although we show the cellular events leading to enamel micropatterning, precise molecular cues that orchestrate directed cell migration still need to be determined.

## Materials and methods

### Animals

The *Sox10-iCreER*^*T2*^*/R26R-Confetti* (*Sox10*^*CreERT2*^*/R26*^*Confetti*^) and *Sox10-iCreER*^*T2*^*/R26R-ZsGreen1* (*Sox10*^*CreERT2*^*/R26*^*ZsGreen1*^) mice were obtained by cross-breeding *CBA;B6-Tg(Sox10-icre/ERT2)388Wdr/J* mice with *B6.129P2-Gt(ROSA)26Sor*^*tm1(CAG-Brainbow2.1)Cle*^*/J* (The Jackson Laboratory (JAX) strain #017492) and *B6.Cg-Gt(ROSA)26Sor*^*tm6(CAG-ZsGreen1)Hze*^*/J* (JAX strain #007906) mouse strains, respectively.^66–68^ All experimental procedures were approved by the Ministry of Education, Youth and Sports of Czech Republic (MSMT-8360/2019-2, MSMT-9231/2020-2). All mice were housed in the conditions of 18–23 °C room temperature, 40%–60% air humidity, 12-h light/dark cycle, and had unlimited access to food and water. Animals were genotyped using endpoint PCR with transgene-specific primers. For the genotyping of the *Sox10*^*CreERT2*^, the following primer sequences were used: GAGGGACTACCTCCTGTACC (Forward primer), TGCCCAGAGTCATCCTTGGC (Reverse primer). Other alleles were genotyped using primer sequences provided by the mouse strain supplier (JAX, USA). Adult (>2 months old), female and male mice with suitable genotypes (carrying the *Sox10-iCreER*^*T2*^ construct, and either *R26R-Confetti* in a homozygous state or *R26R-ZsGreen1* in a heterozygous state) were injected intraperitoneally with 5 mg of tamoxifen (T5648, Sigma-Aldrich, USA) dissolved in corn oil (C8267, Sigma-Aldrich, USA) to induce CreERT2-mediated recombination, and thus the expression of reporter constructs. Animals were euthanized by a combination of isoflurane (sold as Aerrane, 4DG9623 by Baxter S.A., Belgium) overdose and cervical dislocation. Wild-type adult *C57BL/6* mice were used for in situ hybridization and immunostaining.

### Confocal microscopy

To prepare samples for confocal microscopy, dissected mandibles and maxillae were briefly washed in PBS (pH 7.4) and fixed in a 4% solution of paraformaldehyde in PBS (pH 7.4) for 5 to 6 h (for cryosections), 24 h at 4 °C (for paraffin sectioning and immunostaining) or 24 h at room temperature (for RNAscope™). After fixation, the tissues were decalcified in 10% ethylenediaminetetraacetic acid (EDTA, pH 7.4) for 10 days (for cryosections) or 14 days (for paraffin sectioning) at 4 °C while rotating on a tube roller. For cryosections, samples were then incubated in a 30% solution of sucrose in PBS overnight at 4 °C. The following day, tissue samples were carefully washed twice in Tissue-Tek® O.C.T. Compound (Sakura Finetek, USA; sold as 4583 by Medesa, Czech Republic) and embedded into fresh O.C.T. The embedded samples were then stored frozen at −20 °C until sectioning. Sectioning was performed using Leica CM1860 cryostat (Leica Biosystems, Germany) at a thickness of 14 to 300 μm. For paraffin sectioning, samples were dehydrated in series of ethanol and xylene, embedded in paraffin and then sectioned at a 5 μm thickness on a Leica RM2245 microtome (Leica Biosystems, Germany). All sections were transferred onto SuperFrost® Plus microscope slides (Thermo Fisher Scientific, USA; sold as 631-9483 by VWR International). All tissue sections were stained with 4′,6-diamidino-2-phenylindole (DAPI) (D9542, Sigma-Aldrich, USA). Zeiss LSM 880 laser scanning confocal microscope (Carl Zeiss AG, Germany) controlled via ZEN black (ver. 2.3 SP1, Carl Zeiss AG, Germany) was used to image all sections.

### Immunostaining, in situ hybridization

For the detection of calbindin 1 (CALB1), an anti-CALB1 D-28k rabbit polyclonal antibody (1:500) was used (CB-38a, Swant, Switzerland). This antibody was coupled with a donkey-anti rabbit IgG Alexa Fluor™ 647 secondary antibody (A31573, Thermo Fisher Scientific, USA). RNAscope™ Multiplex Fluorescent v2 Assay (323100, Advanced Cell Diagnostics, USA) was used for in situ hybridization according to the manufacturer’s manual with the following probes: Sox2 (401041-C2, Advanced Cell Diagnostics, USA) and Sox10 (435931, Advanced Cell Diagnostics, USA). TSA Vivid™ Fluorophore Kit 570 (7526, Tocris Bioscience, UK) and TSA Vivid™ Fluorophore Kit 650 (7527, Tocris Bioscience, UK) were used for signal amplification.

### Tissue clearing

For samples sectioned at a thickness of 50 μm and greater, tissue clearing was used. Tissue clearing was performed using the Clear, Unobstructed Brain/Body Imaging Cocktails and Computational analysis (CUBIC) protocol.^69^ Clearing solutions were prepared according to the original protocol, albeit further steps were modified in the following manner. Tissue sections of 50 μm were incubated in the CUBIC-1 solution for 30 min, then washed in the CUBIC-2 solution 3 times for 5 min each time. After that, sections were incubated in the CUBIC-2 solution for at least two hours, and then mounted in CUBIC-2 for further storage and imaging.

For tissue sections with a thickness greater than 50 μm, sections were carefully removed from the microscope slides and placed into TPP® 6-well tissue culture plates (Z707767, Sigma-Aldrich, USA) containing the CUBIC-1 solution and DAPI and incubated on a shaker overnight at room temperature. Next, the sections were washed in fresh CUBIC-1 solution twice for 15 min each time, and then incubated in the CUBIC-2 solution for 1 to 2 h. Finally, the sections were carefully transferred onto new microscope slides and embedded into a new CUBIC-2 solution. In order for the cover glass to lay straight on top of the tissue sections, custom 3D-printed plastic frames (spacers) were placed between the microscope slide and the cover glass. The spacers were designed using Tinkercad software (Autodesk, Inc., USA) as a hollow rectangle with the following dimensions: 53 mm (total length) x 23 mm (total width) x 0.5 mm (thickness). The sides were each 1 mm wide. These spacers were then printed in plastic using Prusa i3 MK3S+ 3D printer (Prusa Research a.s., Czech Republic).

### Live imaging

For live imaging experiments, mandibles were quickly dissected from mice and placed into a Petri dish with cold Hanks’ Balanced Salt solution (H6648, Sigma-Aldrich, USA). Incisors were then carefully dissected out of mandibles under a Leica EZ4 stereomicroscope (Leica Microsystems GmbH, Germany) using a no. 11 scalpel (03350, Jai Surgicals Ltd., India) to carefully break the bones surrounding them, as depicted in.^70^ For live imaging experiments conducted using confocal microscopy, samples were mounted onto an ibiTreat 8-well μ-slide (80826, Ibidi, Germany) using a drop of a 3% solution of low gelling temperature agarose (A9414, Sigma-Aldrich, USA) in 3:1 parts of CUBIC-2:PBS, and submerged in imaging medium pre-warmed to 37 °C. For live imaging in a light sheet microscope, samples were mounted directly into the microscope chamber, which contained the pre-warmed imaging medium. The imaging medium was composed as follows (volume percentage): Gibco™ DMEM, high glucose, no glutamine, no phenol red (31053028, Thermo Fisher Scientific, USA), 10% tetracycline free fetal bovine serum (FB-1001T, Biosera, France), 1% penicillin-streptomycin solution (XC-A4122, Biosera, France), 1% Gibco™ GlutaMAX™ supplement (35050061, Thermo Fisher Scientific, USA), 1% Gibco™ MEM non-essential amino acids solution (11140035, Thermo Fisher Scientific, USA). The environment of the microscope chamber was heated to 37 °C and set to contain 5% of CO_2_, the sample was kept submerged in a pre-warmed live imaging medium for the whole duration of data acquisition. Zeiss LSM 880 laser scanning confocal microscope (Carl Zeiss AG, Germany) and Zeiss Lightsheet 7 light sheet fluorescent microscope (Carl Zeiss AG, Germany) were used for imaging, operated via ZEN black (ver. 2.3 SP1, Carl Zeiss AG, Germany). Imaging duration varied from 3 to 12 hours per session. The samples were imaged in equal intervals ranging from 5 to 15 minutes depending on the experiment. For all samples and at each time point, a Z-Stack of an appropriate thickness was acquired, which later enabled the 4D reconstruction of the imaged samples in Imaris (ver. 9.8.2, Oxford Instruments, United Kingdom).

### Data analysis

ZEN blue (ver. 3.7.97.01000, Carl Zeiss AG, Germany) was used for deconvolution, stitching of the images acquired using the Tile Scan function, to perform Dual Side Fusion of data acquired on the light sheet microscope, and for image export. Imaris (ver. 9.8.2, Oxford Instruments, United Kingdom) was used to visualize the samples in 3D space, view and export live imaging time lapses and maximum intensity projection images. Imaris was also used to minimize the effects of sample drift observed in live imaging experiments, by tethering the data to a manually created Reference frame. Briefly, regions of the image, which showed no movement relative to other parts of the tissue between different time points, were manually selected and set as reference points in every third time point. The remaining reference points were automatically extrapolated by Imaris, their accuracy was then manually validated and adjusted if necessary.

Signal quantification was performed using the Measure function in ImageJ (ver. 1.53 v).^71^ First, areas for measuring were manually drawn onto the images (Fig. S2). The fluorescence channels were then split into separate images, and the resulting image containing only the *ZsGreen1* channel was converted into an 8-bit format. A threshold was then applied to the 8-bit image. The same threshold was applied to all images. Finally, the percentage of the manually selected area that was covered with the *ZsGreen1* signal was analyzed using the built-in Measure function.

### Statistical analysis

The measured values were plotted as a box and whiskers graph using Prism software (ver. 9.5.1, GraphPad Software LLC, USA). The central lines on the graph represent median values and the interquartile range (from the 25^th^ to the 75^th^ percentile). All technical replicates are shown as individual data points, with different symbols designating different mice in each timepoint.

Statistical significance of the acquired results was measured using Prism software (ver. 9.5.1, GraphPad Software LLC, USA). To showcase the increase in *ZsGreen1*-positive area within the analyzed ROIs with increasing lineage tracing duration, statistical analyses between subsequent tracing timepoints (groups of samples) were performed. First, tests of normal distribution were performed: D’Agostino & Pearson, Anderson-Darling, Shapiro-Wilk and Kolmogorov-Smirnov tests (*P* < 0.05) were used. Based on the results, parametric tests were chosen for analyses in which pairs of groups of samples were compared (specifically, groups with 1 day, 3 days, 7 days, 14 days and 1 month of lineage tracing), as all of these groups passed normality tests. Next, *F*-test (*P* < 0.05) was conducted to compare the standard deviation variance in these five sample groups. Based on the results, either an unpaired two-tailed *t*-test (*P* < 0.05), or an unpaired two-tailed *t*-test with Welch’s correction (*P* < 0.05) was used. The latter test was used for comparison of sample groups with significantly different (*P* < 0.05) standard deviation variance (groups with 1 day and 3 days of lineage tracing). A nonparametric Kruskal–Wallis test (*P* < 0.05) was undertaken to compare four groups of samples, the two of which (groups with 3 months and 6 months of lineage tracing) did not pass the normality test.

## Supplementary information

Supplementary material

Supplementary Movie 1

Supplementary Movie 2

## Data Availability

The authors declare that all data supporting the conclusions of this study are present within the paper and its Supplementary Materials.
