# Supplementary material for: Enamel decussation pattern originates from directional sliding of ameloblasts
Source: Int J Oral Sci. 2026 Feb 3;18:7. doi: 10.1038/s41368-025-00412-5 (PMC12864829; doi:10.1038/s41368-025-00412-5)
Supplement: Supplementary file 1 — Supplementary material [file 41368_2025_412_MOESM1_ESM.pdf]

Supplementary Information for

Enamel Decussation Pattern Originates from Directional Sliding of Ameloblasts

Vladislav Rakultsev[1,2], Josef Lavicky[1], Marcos Gonzalez Lopez[1], Klara Cigosova[1], Igor Adameyko[3,4], Jan Krivanek[1*]

[1] Department of Histology and Embryology, Faculty of Medicine, Masaryk University, 62500 Brno, Czech Republic

[2] Buchmann Institute for Molecular Life Sciences (BMLS), Institute of Cell Biology and Neuroscience, Goethe University Frankfurt, 60438 Frankfurt am Main, Germany

[3] Department of Neuroimmunology, Center for Brain Research, Medical University of Vienna, 1090 Vienna, Austria

[4] Department of Physiology and Pharmacology, Karolinska Institutet, 17177 Stockholm, Sweden

Authors' email addresses:

Vladislav Rakultsev: rakultsev@mail.muni.cz

Josef Lavicky: josef.lavicky@med.muni.cz

Marcos Gonzalez Lopez: marcos.gonzalez.lopez@med.muni.cz

Klara Cigosova: klara.cigosova@med.muni.cz

Igor Adameyko: igor.adameyko@meduniwien.ac.at

* Correspondence: jan.krivanek@med.muni.cz, tel. +420 549 49 5112.

Figure S1

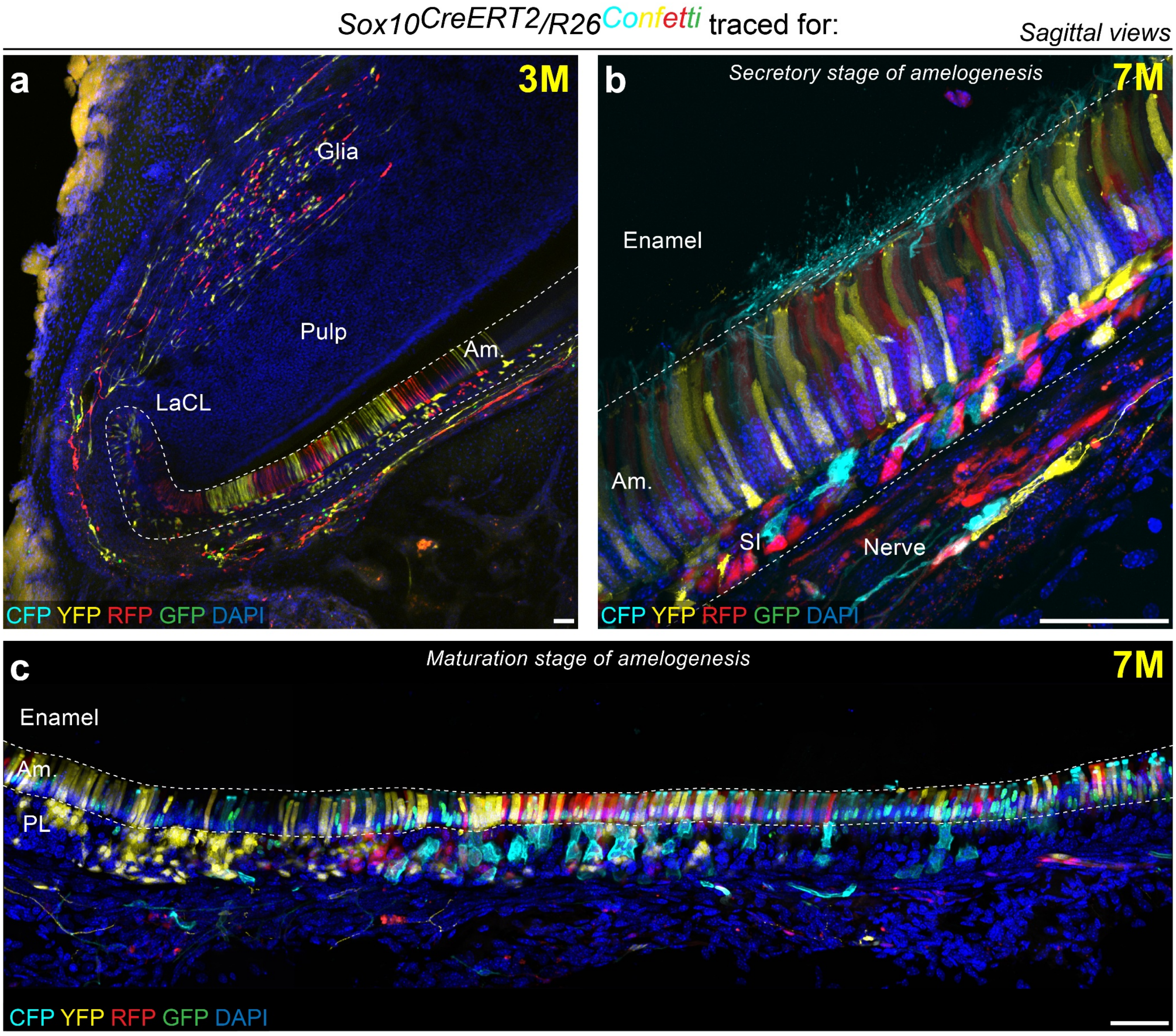

Figure S1. Lineage tracing of *Sox10*+ progenitor cells in mouse incisors

(a–c) Lineage tracing of *Sox10*+ progenitor cells in incisors of $Sox10^{CreERT2}/R26^{Confetti}$ mice shows lineage-traced cells across all layers of the dental epithelium at secretory and maturation stages of amelogenesis and in glial cells, as observed on sagittal tissue sections. Traced cells are present in the labial cervical loop (LaCL), single layers of preameloblasts and ameloblasts, stratum intermedium (SI), stellate reticulum, papillary layer (PL), as well as glia. Section thickness: (a) 200 µm, (b) and (c) 50 µm. Scale bars: 50 µm. (Am. – ameloblasts, LaCL – labial cervical loop, M – months of lineage tracing, PL – papillary layer, SI – stratum intermedium).

Figure S2

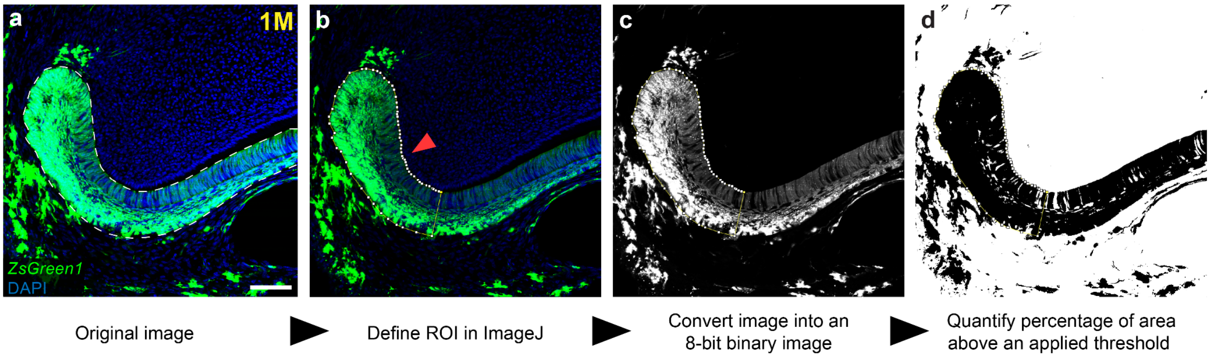

Figure S2. Image analysis pipeline used for the evaluation of *Sox10*+ cell expansion in the amelogenic epithelium

(a–d) Analysis pipeline used for the evaluation of expansion of lineage-traced *Sox10*+ cell progeny in the amelogenic epithelium (lingual cervical loop) of *Sox10^CreERT2^/R26^ZsGreen1^* mouse incisors. Dashed lines in (a) delineate dental epithelium. Regions of interest (ROI) were manually defined in ImageJ, as indicated with an arrowhead in (b). The original image was then split into separate fluorescent channels, and the *ZsGreen1* channel was converted into a 8-bit binary image (c). The created ROI was then overlayed on top of the converted image. Lastly, an identical signal threshold was applied to all analyzed images, and the percentage of the ROI that contains the *ZsGreen1* signal (after conversion, with the applied threshold) was quantified. Scale bar: 30 µm. (M – months of lineage tracing, ROI – region of interest).

Figure S3

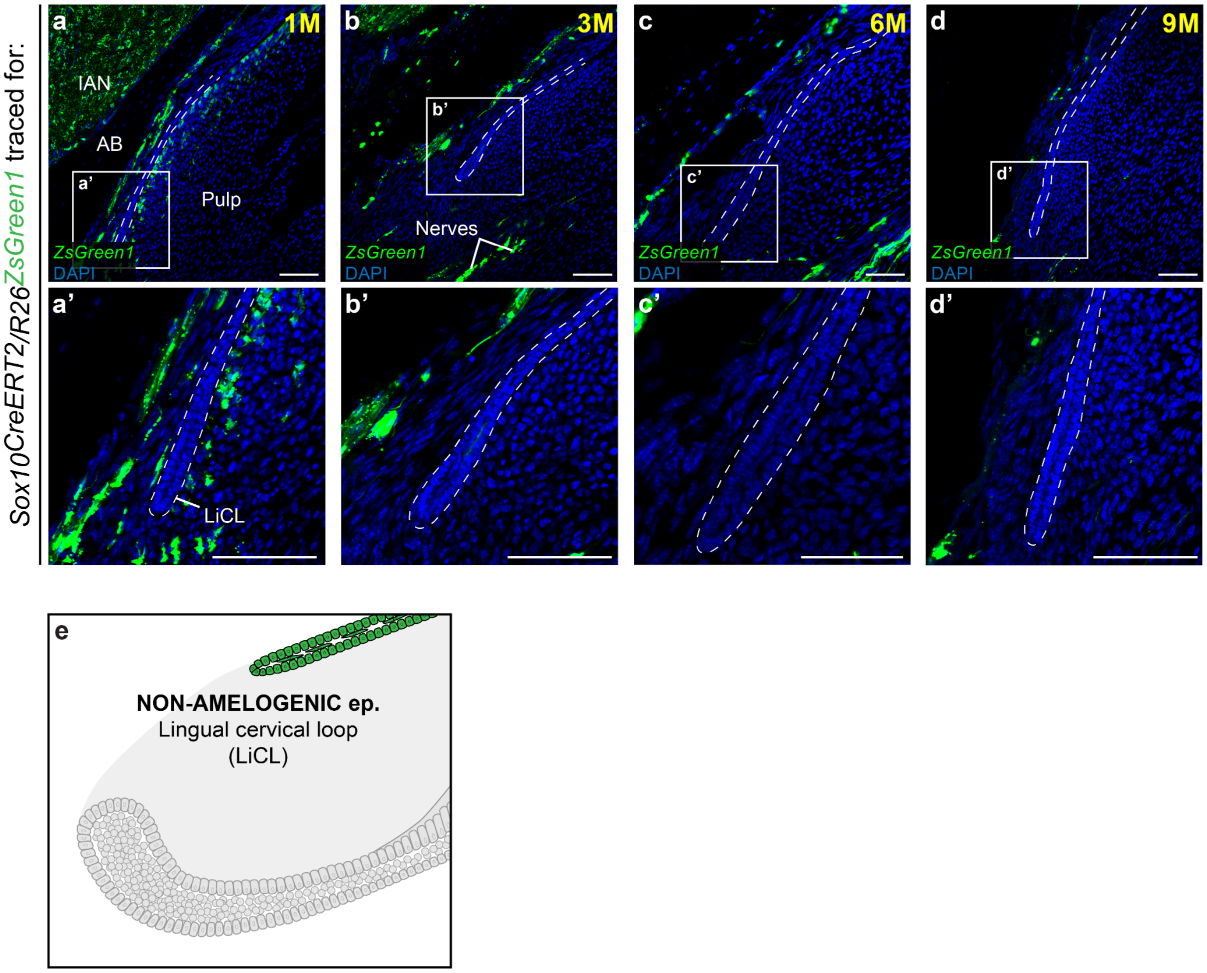

Figure S3. Absence of *Sox10*+ progenitor cells in the non-amelogenic dental epithelium

(a–d) Lineage tracing of *Sox10*+ progenitor cells in $Sox10^{CreERT2}/R26^{ZsGreen1}$ mouse incisors on 14 µm sagittal sections reveals that no traced cells are present in the non-amelogenic lingual cervical loop (LiCL) even after long-term (9 months) lineage tracing. (a'–d') Detailed sagittal views of the LiCL region showing no traced cells inside the LiCL. (e) Schematic drawing of the apical part of the incisor in a sagittal view, depicting non-amelogenic epithelial cells of the LiCL (green). Scale bars: 30 µm. (AB – alveolar bone, IAN – inferior alveolar nerve, LiCL – lingual cervical loop, M – months of lineage tracing).

Figure S4

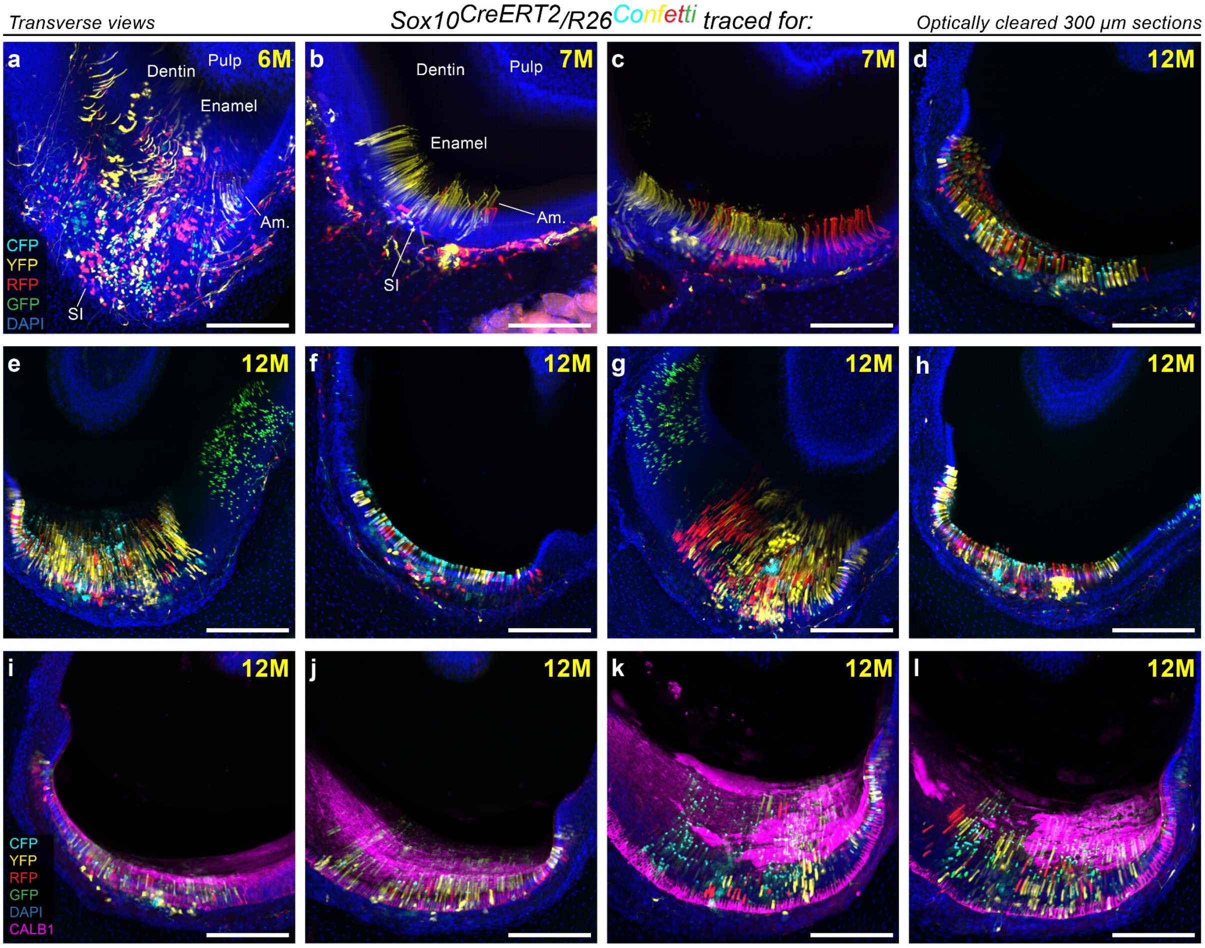

Figure S4. Distribution of clonal clusters of ameloblasts on transverse slices of incisors

(a–l) Long-term lineage tracing of *Sox10*+ progenitor cells on 300 µm optically cleared transverse sections of *iCreER*^{T2}*/R26R-Confetti* mouse incisors shows groups of ameloblasts of mixed origin, in which cells labeled with different fluorescent proteins are combined together. Sections (i)–(l) were additionally immunostained with an anti-*CALB1* antibody to label the ameloblast cell layer. Scale bars: 150 µm. (Am. – ameloblasts, M – months of lineage tracing, SI – stratum intermedium).

Figure S5

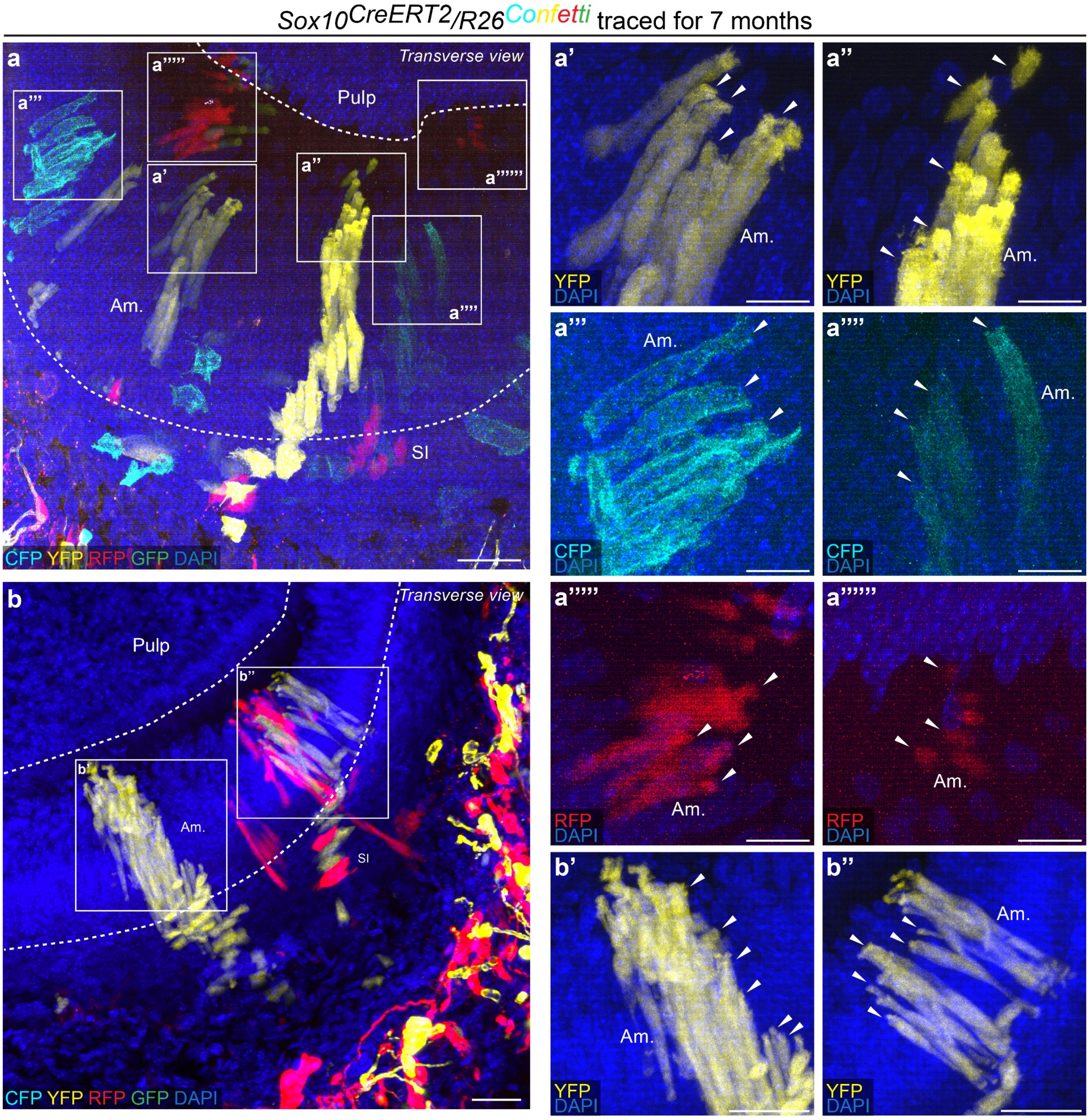

Figure S5. Spatial distribution of clonal clusters of ameloblasts

(a and b) Confocal microscopy images of sections of lineage-traced *Sox10*^{CreERT2}*/R26*^{Confetti} mouse incisors in a transverse plane show ameloblasts labeled with a common fluorescent protein split into similarly sized groups. (a'–a'''''' and b'–b'') Detailed view of grouped ameloblasts shown in (a) and (b), expressing a single shared fluorescent protein per group. Note how Tomes' processes (arrowheads) of ameloblasts are oriented towards the point of origin of respective cells. Section thickness: (a) 300 µm, (b) 50 µm. Scale bars: 10 µm in (a), (a')–(a''''''), 30 µm in (b), (b') and (b''). (Am. – ameloblasts, SI – stratum intermedium).

Supplementary movie S1. Live imaging of ameloblast migration

Live imaging time-lapse movie of *Sox10*^{CreERT2}*/R26*^{Confetti} mouse incisor tissue explant following a single traced (RFP-labeled) ameloblast clone shows the possible directions of movement of ameloblasts within a single clone, possibly leading to the formation of a decussation pattern. Some groups of cells migrate in opposite directions away from the line of the split.

Supplementary movie S2. Live imaging of cell migration in the labial cervical loop

Live imaging of the labial cervical loop of *Sox10*^{CreERT2}*/R26*^{Confetti} mouse incisors shows cells migrating from the outer enamel epithelium layer into the stellate reticulum**.**
